# [194 + 2T>C;−215G>A] mutation of *SPINK1* gene is associated with fibrocalculous pancreatic diabetes: A rare case report

**DOI:** 10.1097/MD.0000000000044613

**Published:** 2025-10-03

**Authors:** Yan Li, Qing-Hui Liao, Li-di Zhong, Qin Du, Yao-Ming Xue, Yu-Hua Chen, Ping Liu, Min Liu

**Affiliations:** a Endocrinology and Metabolism Department of The Second Affiliated Hospital, School of Medicine, The Chinese University of Hong Kong, Shenzhen and Longgang District People’s Hospital of Shenzhen, Shenzhen, Guangdong, PR. China; b Endocrinology and Metabolism Department of Nanfang Hospital, Southern Medical University, Guangzhou, Guangdong, PR. China.

**Keywords:** FCPD, fibrocalculous pancreatic diabetes, pancreatic calcifications, *SPINK1*, the serine protease inhibitor Kazal type 1

## Abstract

**Rationale:**

Fibrocalculous pancreatic diabetes (FCPD) is a unique form of diabetes reported from tropical and subtropical regions, associated with both endocrine and exocrine disease of the pancreas, frequently misdiagnosed, resulting in delayed therapy to patients.

**Patient concerns:**

A now 41-year-old Chinese male had a 11-year history of dry mouth, polydipsia, polyuria, progressive weight loss, recurrent abdominal bloating and diarrhea.

**Diagnoses:**

He were misdiagnosed as Type 1 diabetes mellitus, due to many similar characteristic features between FCPD and T1DM.The patient received whole gene sequencing. Two mutations were identified, the pathogenic splicing mutation c.194 + 2T > C (IVS3 + 2T > C) and a Variant of Undetermined Significance c.-215G > A.

**Interventions:**

He underwent extracorporeal shock wave lithotripsy combined with the conventional eneoscopic retrograde cholangio pancreatography and received addition of multiple daily insulin injections therapy.

**Outcomes:**

He reported reduced gastrointestinal symptoms. He and his relatives’s long-term follow-up management was conducted.

**Lessons:**

Clinically, FCPD deserves more attention than it currently receives, because of its unique clinical features and management strategies, and its propensity to develop diabetes-related vascular complications and pancreatic adenocarcinoma.

## 1. Introduction

Fibrocalculous pancreatic diabetes (FCPD) first reported by Zudeima in 1959.^[1]^ It is a unique form of diabetes reported from tropical and subtropical regions (Asia, Africa, and South America),^[2–6]^ it caused by chronic nonalcoholic calcific pancreatitis characterized by irreversible destruction of the pancreatic parenchyma, fibrosis, calcification, and pancreatic ductal calculi, resulting in exocrine pancreatic insufficiency and progressive endocrine dysfunction.^[7]^ The main clinical manifestations are abdominal pain, chronic pancreatitis and diabetes mellitus caused by lack of insulin secretion, accompanied by different degrees of malnutrition. The etiology and pathogenesis of FCPD are unclear, with genetic predisposition and familial aggregation, and associated with malnutrition, cassava consumption, and micronutrient deficiencies. FCPD is different from other diabetes mellitus and requires combined medical and surgical treatments. FCPD increases the risk of pancreatic cancer, and regular screening for pancreatic malignant tumors is needed. The prevalence of FCPD is 0.36% in patients with diabetes mellitus.^[8]^ Although the prevalence of diabetes mellitus in Chinese adults is 14.92%, only a few reports have been published on FCDP patients to date. Most of them were misdiagnosed as Type 1 diabetes mellitus (T1DM),^[8]^ due to many similar characteristic features between FCPD and T1DM.

## 2. Case presentation

The now 41-year-old male was born in the Shiyan City of Hubei Province. And he has lived in Shenzhen of Guangdong Province for many years. He had a 11-year history of dry mouth, polydipsia, polyuria, progressive weight loss (15 kilograms), recurrent abdominal bloating and diarrhea. He was being treated as a patient with T1DM with onset of ketoacidosis and less residual beta cell function. He received basal-bolus insulin and mealtime rapid acting insulin therapy. However, the patient was reluctant to inject 4 times per day in long term. He stopped exogenous insulin and tried oral hypoglycemic agents (Metformin, Sitagliptin, Dapagliflozin). At his first visit to our hospital in March 2022, he continued to have poor glycemic control, but without Ketosis or ketoacidosis.

He had no history of consumption of alcohol or cassava, gall stone disease, or hepatitis, and no family history of pancreatic disease or diabetes mellitus. His parents were not consanguineous marriage.

On examination, his blood pressure was 102/67 mm Hg, pulse rate 75/min, respiratory rate 17/min and pulse oxygen saturation 100%. His body mass index was 22.57 kg/m^2^. His neurological, cardiorespiratory and abdomen examinations were normal.

**Figure 1.**
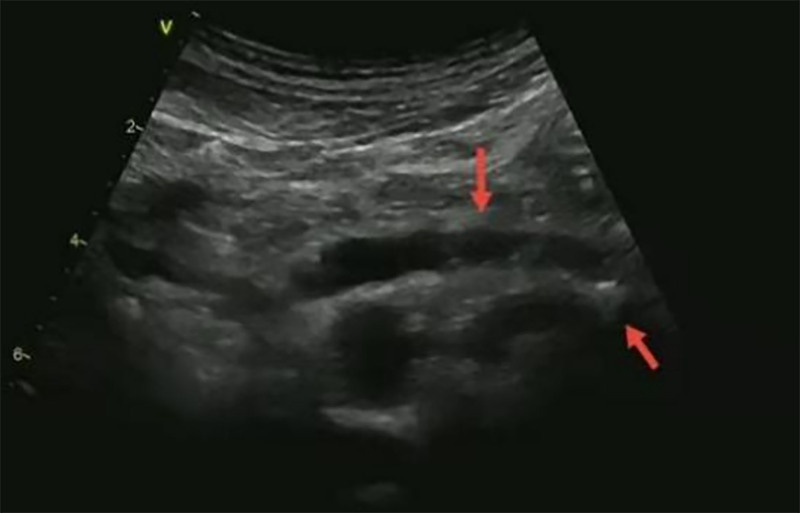
Ultrasound of abdomen showed dilated main pancreatic duct and hyperechoic dots of the pancreatic tail.

**Figure 2.**
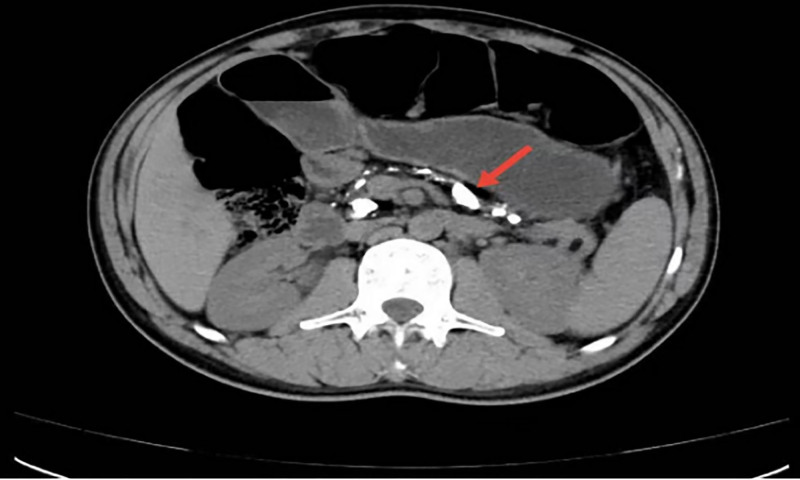
Plain computed tomography abdomen showed multiple calcifications involving the head, body and tail of pancreatic parenchyma.The dilated main pancreatic duct was also seen. (A) Axial plane. (B) Coronal plane.

The haematological investigations was planned. The routine blood test, urinalysis, lipid profile, renal and liver function tests were normal. The stool analysis revealed no fat globules. The results of other biochemical indicators are shown in Table 1. His funduscopy, the bilateral carotid ultrasonography examination, electromyography were normal. Ultrasound of abdomen showed dilated main pancreatic duct and hyperechoic dots of the pancreatic tail (Fig. 1). Plain computed tomography abdomen showed multiple calcifications involving the head, body and tail of pancreatic parenchyma (Fig. 2). This presentation met the diagnostic criteria for FCDP. This case had no macrovascular or microvascular complications of diabetes mellitus.

**Table 1:** The results of clinical biochemical tests.

Items	Results	Reference value
Fasting sugar	5.38	3.89–6.11 mmol/L
Fasting C-peptide	0.23	0.30–3.73 ng/mL
Post prandial sugar	5.46	<7.80 mmol/L
Post prandial C-peptide	0.18	0.30–3.73 ng/mL
Hemoglobin A1c	8.3	4.0%–6.0%
Markers of immune destruction of beta Cells (insulin, glutamic acid decarboxylase, and tyrosine phosphatase IA-2 antibodies)	Negative	Negative
Serum calcium	2.37	2.10–2.55 mmol/L
Parathyroid hormone	2.77	1.32–9.52 pg/mL
Serum vitamin D	12.7	9.3–47.9 ng/mL
Serum Tumor Markers	negative	Carcinoembryonic antigen: 0–5 ng/mL
		Carbohydrate antigen 125: 0–35 U/mL
		Carbohydrate antigen 199: 0–25 mU/mL
		Alpha fetoprotein: 0–20 ng/mL
24 h urinary protein	185	<150 mg/24h

**Figure 3.**
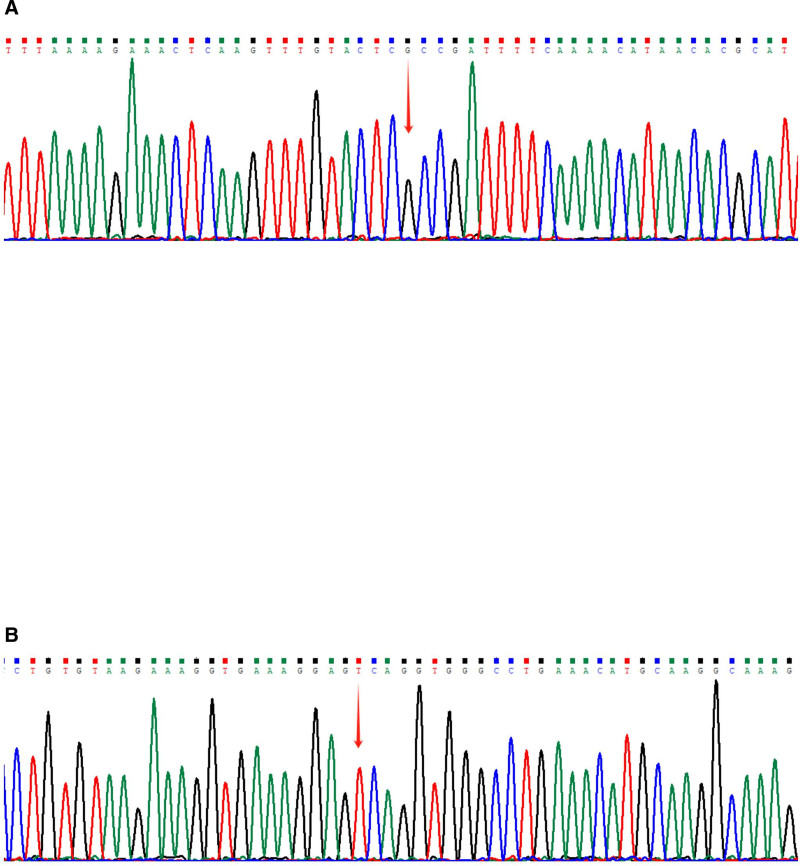
Results of patient genome sequencing data. (A) *SPINK1*: c.194 + 2T > C (IVS3 + 2T > C). (B) *SPINK1*: c.-215G > A. *SPINK1* = serine protease inhibitor Kazal type 1

Based on the results, whole gene sequencing was performed. We collected peripheral blood from this patient, extracted the genomic DNA of the samples sent for examination, fragmented, ligated the junctions, amplified and purified the DNA, broke the DNA and prepared libraries, and then captured and enriched the DNA in the exons and adjacent shear regions of the target genes by the Roche KAPA HyperExome microarray, and finally detected the mutations by using the MGISEQ-2000 and DNBSEQ- T7 sequencing platform for variant detection. Sequenced fragments were aligned to the UCSC hg19 human reference genome by Burrows-Wheeler Aligner to remove duplicates. Exon level copy number variant detection was performed using ExomeDepth. Variants were categorized for pathogenicity according to the American College of Medical Genetics and Genomics and the American Molecular Pathology Society guidelines for interpretation of sequence variants, as well as the ClinGen Task Force for Interpretation of Sequence Variants and the British Academy of Clinical Genome Sciences. Two mutations were identified, the pathogenic splicing mutation NM_003122.3 (Serine protease inhibitor Kazal type 1, *SPINK1*): c.194 + 2T > C (IVS3 + 2T > C) and a Variant of Undetermined Significance NM_ 003122.3 (*SPINK1*): c.-215G > A (Fig. 3). This patient with FCDP was compound homozygous for the [194 + 2T > C; −215G > A;] mutation.

Management: 1) Endocrine functions of the pancreas and glycaemic control. Based on the FCPD diagnosis and a great degree of impairment in insulin secretion,^[9]^ this patient’s management protocol was modified with addition of multiple daily insulin injections therapy, withdrawing oral hypoglycemic agents. His glycaemic control improved. Management of pain. This patient with large pancreatic stones were quite difficult to be removed by conventional eneoscopic retrograde cholangio pancreatography. He underwent extracorporeal shock wave lithotripsy combined with conventional eneoscopic retrograde cholangio pancreatography. He reported reduced gastrointestinal symptoms. Exocrine functions of the pancreas. He was also treated with pancreatic enzyme replacement therapy. Life style interventions. Low-fat diet, avoid alcohol, exercise, blood glucose monitoring, maintaining good hydration, avoid stress, a daily vitamin D supplement. Genetic counseling. We recommended genetic testing for *SPINK1* variants in first-degree relatives of our patient.

## 3. Discussion

Etiopathogenesis of FCPD is multifactorial and not yet well determined, with cassava intake, micronutrient deficiencies, oxidant stress, autoimmune factors being implicated.^[7]^ Diagnostic criteria for FCPD.^[10]^ occurrence in a “tropical” country or region. Diabetes by WHO Study Group criteria.^[11]^ Evidence of chronic pancreatitis: pancreatic calculi on X-ray or at least 3 of the following: abnormal pancreatic morphology by ultrasonography, chronic abdominal pain since childhood, steatorrhoea, abnormal pancreatic function test. Absence of other causes of chronic pancreatitis. i.e. Alcoholism, hepatobiliary disease or primary hyperparathyroidism etc. On the basis of clinical and radiological findings, this patient’s diagnosis of FCPD was made over 11 years later.

We have carefully analyzed the causes of misdiagnosis so as to improve diagnosis accuracy of the disease. **The different modes of onset.** Generally, diabetic ketoacidosis don’t commonly occur in FCPD, this is attributed to partial beta cell destruction with some insulin secretory activity present, reduced glucagon reserve because of alpha cell destruction, and finally, reduced availability of non-esterified fatty acids which are a substrate for ketogenesis.^[2]^ We reported a FCPD patient with ketoacidosis, it is concluded that ketosis is not an exclusion criterion for FCPD. Indeed, hyperglycemia, islet beta cell dysfunction, and especially pancreatic calculi on imaging play vital roles in diagnosis. **Limitations of pancreatic ultrasonography.** It is generally believe that FCPD is a secondary form of diabetes mellitus due to chronic calcific pancreatitis. So how to explain the pancreatic ultrasound was normal when he was confirmed diabetes mellitus? At that time, the definition and resolution of ultrasonic imaging system were limited. Moreover, our patient had symptom of recurrent abdominal bloating, gastrointestinal gas interference pancreas showed. In addition, the accuracy of ultrasound diagnosis relied on the experience of the doctors, the results affected by the subjective factors of the doctors in different degrees. Unfortunately, this patient did not have abdominal X-ray or CT scan at his first visit, so we could not completely exclude that he had been in combination with pancreatic calcification or stones at that time. **Lack of recognition.** Although FCPD has classical clinical symptoms, it is easy to be misdiagnosed because of lack of recognition, most of patients may be misdiagnosed as T1DM.

The human *SPINK1* (also called tumor‐associated trypsin inhibitor) gene is located on chromosome 5 and consists of approximately 7.5 kilobases, including 4 exons. The *SPINK1* protein is 56 amino acids long and contains 6 cysteine residues.^[12]^ Physiologically, *SPINK1* is secreted by pancreatic acinar cells as the first line of defense against premature activation of trypsinogen in the acinar and pancreatic duct. *SPINK1* regulates normal pancreas development.^[13]^ *SPINK1* mutations might result in altered interaction between *SPINK1* and trypsin, thus affecting the protease/ antiprotease balance within the pancreas.^[14,15]^ We have compared other genes associated with FCPD in Table 2.^[16–21]^

**Table 2:** Other related genes of FCPD.

Symbol	Variation	Types	Expression level	Function
Chymotrypsin C, *CTRC*	Susceptibility factor	p.A73T (severe secretion defect)	↓	The mutations caused loss of *CTRC* function by various mechanisms.
		p.K247_R254del (degradation)		
		p.R254W (degradation)		
		p.V235I (reduced activity)		
Cathepsin B, *CTSB*	The candidate gene	p.L26V and p.S53G affected the transport of *CTSB*).	↓	Mutations in the *CTSB* gene may be involved in inappropriate localization or premature activation of trypsinogen and lead to pancreatitis.
Serine Protease 1, *PRSS1*	–	The p.R122C and p.R122H mutations prevented *CTRC*-mediated trypsinogen degradation.The p.N29I mutation increased trypsinogen autoactivation.	↓	*PRSS1* mutations stimulated activation of cationic trypsinogen.
The calcium-sensing receptor, *CASR*	–	p.R990G, p.A986S increased chronic pancreatitis risk.	↓	*CASR* may respond to high calcium concentrations in the juice and prevent stone formation.
The cystic fibrosis transmembrane conductance regulator, *CFTR*	Susceptibility factor	p.F508del mutation (protective variant)	↓	*CFTR* mutations disrupted channel activity or affected membrane levels.
		p.R117H mutation increased risk.		
Serine protease 2, *PRSS2*	–	A protective variant p.G191R.	NA	The mutation introduced a new trypsin cleavage site into anionic trypsinogen, which increased autocatalytic proteolysis and inactivation.

**Figure 4.**
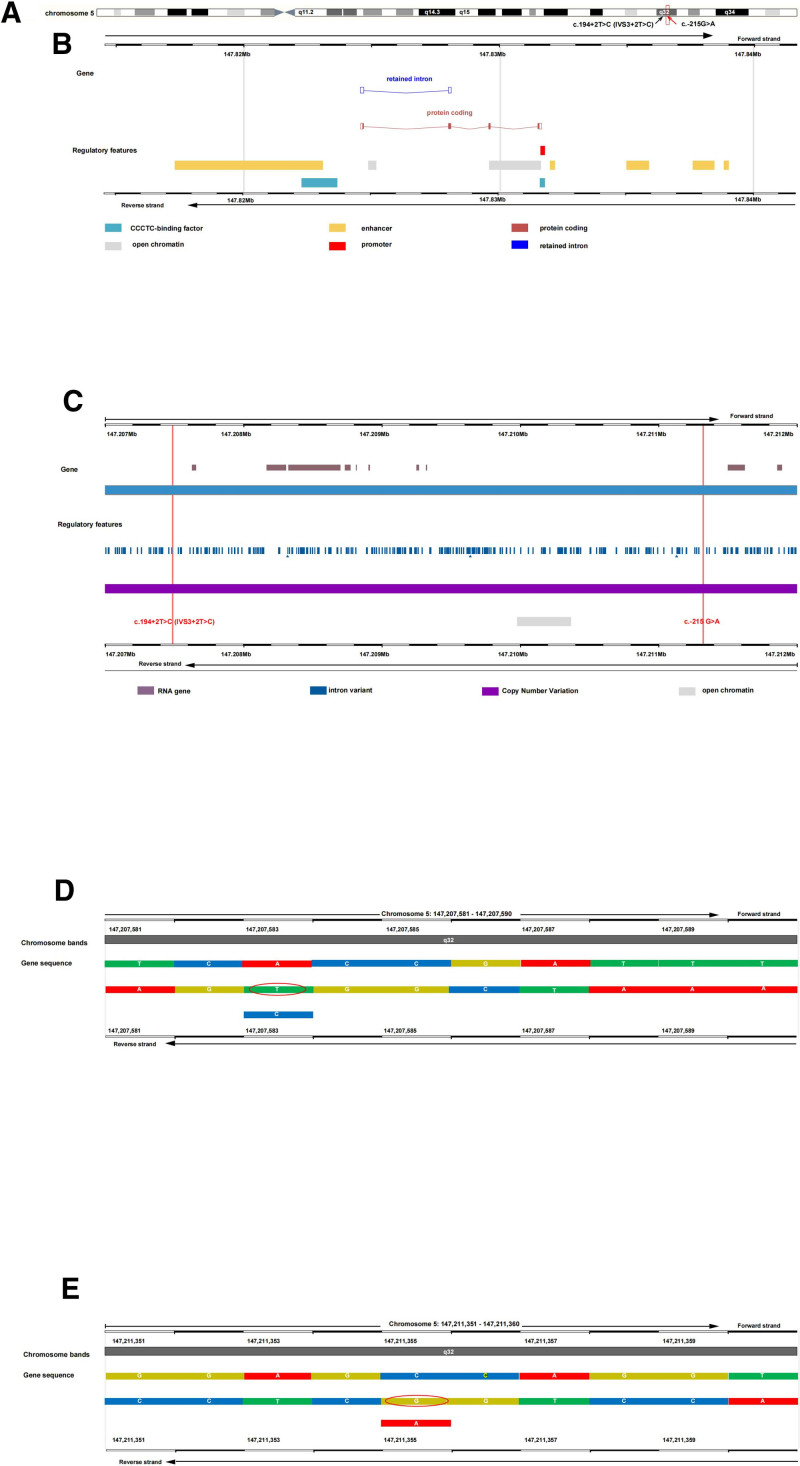
The schematic localization of *SPINK 1* variants. The genomic coordinates are based on human genome build hg19 (GRCh37). (A) Chromosome location of the *SPINK 1, SPINK 1*:c.194 + 2T > C (IVS3 + 2T > C), *SPINK1*: c.-215G > A. (B) Gene structure schematic of *SPINK 1.* (C) Gene structure schematic of *SPINK 1*:c.194 + 2T > C (IVS3 + 2T > C), *SPINK1*: c.-215G > A. (D) The schematic localization of *SPINK1*: c.194 + 2T > C (IVS3 + 2T > C). (E) The schematic localization of *SPINK1*: c.-215G > A. Image source: Chromosome 5: 147,824,572 to 147,831,671 [Internet]. ensembl. [cited 2025 Sep 20]. Available from: Gene: SPINK1 (ENSG00000164266) - Summary - Homo_sapiens - Ensembl genome browser 115https://asia.ensembl.org/Homo_sapiens/Location/View. *SPINK1* = serine protease inhibitor Kazal type 1

The several *SPINK1* variants could lead to FCPD (Table 3).^[22–26]^ The reported prevalence of *SPINK1* gene variations in patients with FCPD ranges from 33% to 46%.^[27]^ Most of these reports on *SPINK1* gene variations in FCPD were from the Indian subcontinent.^[28]^ In Chinese pancreatitis patients, C.194 + 2T > C mutation is most frequently observed and influences the clinical course.^[29]^ Patients carrying the C.194 + 2T > C mutation may less achieve pain relief through endoscopic treatments.^[29]^ They also have a higher risk of endocrine function deterioration.^[29]^ Due to small number of cases, it is unknown whether Chinese ethnicity may be associated with FCPD susceptibility genes.^[8]^ It is the first report of the *SPINK1* [194 + 2T > C;−215G > A;] mutation in FCPD globally (Fig. 4).

**Table 3:** Variants of *SPINK1* gene for FCPD.

rsID	Variation name	Reference/Alternate	Amino acid change	Types	Clinical significance	The function of variants for *SPINK1* protein
rs148954387	NM_001379610..1 (*SPINK1*): c.194 + 2T > C	A/G	–	Splice donor variant	Pathogenic	The c.194 + 2T > C intronic alteration abolished *SPINK1* expression and increased the risk of chronic pancreatitis by diminishing protective trypsin inhibitor levels.^[22]^ The c.194 + 2T > C mutation could predispose patients to earlier onset of pancreatic diabetes. It may preferentially destroy islet cells.^[23]^
rs191068215	NM_003122..5 (*SPINK1*): c.-191-24G > T	C/A	–	5 Prime untranslated region variant, intron variant	Pathogenic	Not available
rs17107315	NM_001379610..1 (*SPINK1*): c.101A > G (p.Asn34Ser)	T/C	N34S	Missense variant	Pathogenic/Likely pathogenic	N34S alteration was seen to increase the susceptibility of *SPINK1* for inactivation by cathepsin L.^[24]^
rs769727763	NM_001379610..1 (SPINK1): c.80G > A (p.Gly27Glu)	C/T	G27E	Missense variant	Uncertain significance	Not Available
rs779832256	NM_003122..5 (SPINK1): c.-147A > G	T/C	–	5 Prime untranslated region variant, genic upstream transcript variant	Uncertain significance	The c.−147A > G disrupted the Hepatocyte Nuclear Factor 1-binding site, and reduced SPINK1 gene expression.^[25]^
rs111966833	NM_001379610..1 (SPINK1): c.163C > T (p.Pro55Ser)	G/A	P55S	Missense variant	Likely benign	The reason for the slightly reduced mRNA expression of the c.163C > T mutant in relation to the wild-type molecule is unclear.^[26]^
rs199929811	NM_001379610..1 (SPINK1): c.88-23A > T	T/A	–	Intron variant	Benign	Not available

FCPD = fibrocalculous pancreatic diabetes, SPINK1 = serine protease inhibitor Kazal type 1.

Kume, K reported the [194 + 2T > C;−215G > A;] mutation is associated with familial and idiopathic chronic pancreatitis in Japan.^[30]^ The c.194 + 2T > C mutation affects the consensus splicing site in intron 3 which leads to skipping of the entire exon 3 where the trypsin binding site is located.^[31]^ The c.-215G > A variant has been seen in the Genome Aggregation Database 148 times with an allele frequency off 1.05e-4. It is in complete linkage disequilibrium with c.194 + 2T > C, any influence of the c.-215G > A variant on *SPINK1* gene expression would be completely masked in vivo by the dramatic effect of the cis-linked c.194 + 2T > C mutation on the mRNA splicing phenotype.^[25]^ But further molecular mechanisms is in necessity to testify that the compound homozygous [194 + 2T > C;−215G > A;] mutation is linked to FCPD and how these mutations influence disease phenotype and progression.

Scholars reported that familial clustering of FCPD and positive association of *SPINK1*^[32]^ points to the possible role of genetic predisposition,^[28]^ thus the gene-sequencing analyses of the patient’s family should be conducted.Importantly, in case of his relatives, having genetic variants should be carefully followed and periodically screened by the clinicians.

## 4. Conclusion

The patient had long-term clinical manifestations of recurrent abdominal bloating, chronic diarrhea, diabetes and pancreatic calcification, it was a typical case of FCPD. However, he had failed to get definite diagnosis for more than 11 years. Clinically, FCPD deserves more attention than it currently receives, because of its unique clinical features and management strategies, and its propensity to develop diabetes-related vascular complications^[33]^ and pancreatic adenocarcinoma.^[34]^

Additionally, we suggest the use of abdominal X-ray or CT scan as an essential diagnostic tool for the suspected cases. Genetic counseling should be provided throughout the entire process. Genetic testing is helpful in the diagnosis and understand the characteristics of gene expression in Chinese patients with FCPD. Furthermore, *SPINK1* is expected to be a marker for the diagnosis, prognosis evaluation, and disease monitoring of FCPD. Gene therapy may become a novel therapeutic target for FCPD.

In conclusion, FCPD patients should be screened regularly (related markers in diabetic complications and pancreatic cancer) and managed by a multidisciplinary team.

## Author contributions

**Conceptualization:** Yan Li, Qing-hui Liao, Min Liu.

**Data curation:** Yan Li, Qing-hui Liao.

**Investigation:** Yan Li, Qing-hui Liao, Qin Du.

**Methodology:** Yan Li, Qing-hui Liao, Li-di Zhong.

**Resources:** Yan Li, Min Liu.

**Writing – original draft:** Yan Li.

**Formal analysis:** Qing-hui Liao.

**Project administration:** Qing-hui Liao, Yao-ming Xue, Yu-hua Chen, Ping Liu, Min Liu.

**Validation:** Qing-hui Liao, Li-di Zhong, Qin Du, Min Liu.

**Writing – review & editing:** Qing-hui Liao, Yao-ming Xue, Yu-hua Chen, Ping Liu, Min Liu.

**Supervision:** Li-di Zhong, Yao-ming Xue, Yu-hua Chen, Ping Liu, Min Liu.

**Visualization:** Li-di Zhong, Qin Du.

**Software:** Qin Du.

**Funding acquisition:** Yu-hua Chen, Min Liu.

## References

[1] Zuidema PJ. Cirrhosis and disseminated calcification of the pancreas in patients with malnutrition. Trop Geogr Med. 1959;11:70–4. 13659585

[2] Barman KK Premalatha G Mohan V. Tropical chronic pancreatitis. Postgrad Med J. 2003;79:606–15. 14654569 10.1136/pmj.79.937.606 PMC1742869

[3] Kamin M Khan SA Rehman T. Images of the month 1: fibrocalculous pancreatic diabetes (FCPD): a rare form of secondary diabetes. Clin Med (Lond). 2021;21:e408–9. 35192485 10.7861/clinmed.2021-0041 PMC8313205

[4] Takasawa K Miyakawa Y Masamune A Kashimada K Shimohira M. Fibrocalculous pancreatic diabetes in a Japanese girl with severe motor and intellectual disabilities. Acta Diabetol. 2016;53:507–10. 26782097 10.1007/s00592-015-0834-9

[5] Kibirige D Kibudde S Mutebi E. Fibrocalculous pancreatic diabetes in a young Ugandan patient, a rare form of secondary diabetes. BMC Res Notes. 2012;5:622. 23126518 10.1186/1756-0500-5-622 PMC3514369

[6] Dani R Penna FJ Nogueira CE. Etiology of chronic calcifying pancreatitis in Brazil: a report of 329 consecutive cases. Int J Pancreatol. 1986;1:399–406. 3681031 10.1007/BF02801872

[7] Kumaran S Unnikrishnan AG. Fibrocalculous pancreatic diabetes. J Diabetes Complications. 2021;35:107627. 32553576 10.1016/j.jdiacomp.2020.107627

[8] Xia F Zhou W Wang B Hu Y. Non-tropical fibrocalculous pancreatic diabetes: case reports and review of recent literature. J Int Med Res. 2020;48:300060520938967. 32691650 10.1177/0300060520938967 PMC7375728

[9] Aiswarya Y Shivaprasad C Anish K Sridevi A Anupam B Amit G. Assessment of insulin sensitivity and secretion in patients with fibrocalculous pancreatic diabetes. Diabetes Metab Syndr Obes. 2019;12:779–88. 31190936 10.2147/DMSO.S204254 PMC6535669

[10] American Diabetes Association Professional Practice Committee. 2. Diagnosis and classification of diabetes: standards of care in diabetes-2025. Diabet Care. 2025;48:S27–49. 10.2337/dc25-S002 PMC11635041 39651986

[11] Alberti KG Zimmet PZ. Definition, diagnosis and classification of diabetes mellitus and its complications. Part 1: diagnosis and classification of diabetes mellitus provisional report of a WHO consultation. Diabet Med. 1998;15:539–53. 9686693 10.1002/(SICI)1096-9136(199807)15:7<539::AID-DIA668>3.0.CO;2-S

[12] Horii A Kobayashi T Tomita N. Primary structure of human pancreatic secretory trypsin inhibitor (PSTI) gene. Biochem Biophys Res Commun. 1987;149:635–41. 3501289 10.1016/0006-291x(87)90415-3

[13] Liao C Wang Q An J. SPINKs in tumors: potential therapeutic targets. Front Oncol. 2022;12:833741. 35223512 10.3389/fonc.2022.833741 PMC8873584

[14] Witt H Luck W Hennies HC. Mutations in the gene encoding the serine protease inhibitor, Kazal type 1 are associated with chronic pancreatitis. Nat Genet. 2000;25:213–6. 10835640 10.1038/76088

[15] Pfützer RH Barmada MM Brunskill AP. SPINK1/PSTI polymorphisms act as disease modifiers in familial and idiopathic chronic pancreatitis. Gastroenterology. 2000;119:615–23. 10982753 10.1053/gast.2000.18017

[16] Beer S Zhou J Szabó A. Comprehensive functional analysis of chymotrypsin C (CTRC) variants reveals distinct loss-of-function mechanisms associated with pancreatitis risk. Gut. 2013;62:1616–24. 22942235 10.1136/gutjnl-2012-303090 PMC3660471

[17] Mayerle J Sendler M Hegyi E Beyer G Lerch MM Sahin-Tóth M. Genetics, cell biology, and pathophysiology of pancreatitis. Gastroenterology. 2019;156:1951–68.e1. 30660731 10.1053/j.gastro.2018.11.081 PMC6903413

[18] Mahurkar S Idris MM Reddy DN. Association of cathepsin B gene polymorphisms with tropical calcific pancreatitis. Gut. 2006;55:1270–5. 16492714 10.1136/gut.2005.087403 PMC1860014

[19] Szabó A Sahin-Tóth M. Increased activation of hereditary pancreatitis-associated human cationic trypsinogen mutants in presence of chymotrypsin C. J Biol Chem. 2012;287:20701–10. 22539344 10.1074/jbc.M112.360065 PMC3370252

[20] Rácz GZ Kittel A Riccardi D Case RM Elliott AC Varga G. Extracellular calcium sensing receptor in human pancreatic cells. Gut. 2002;51:705–11. 12377811 10.1136/gut.51.5.705 PMC1773426

[21] Witt H Sahin-Tóth M Landt O. A degradation-sensitive anionic trypsinogen (PRSS2) variant protects against chronic pancreatitis. Nat Genet. 2006;38:668–73. 16699518 10.1038/ng1797 PMC2746914

[22] Kereszturi E Király O Sahin-Tóth M. Minigene analysis of intronic variants in common SPINK1 haplotypes associated with chronic pancreatitis. Gut. 2009;58:545–9. 18978175 10.1136/gut.2008.164947 PMC2677899

[23] Sun C Liao Z Jiang L. The contribution of the SPINK1 c.194 + 2T>C mutation to the clinical course of idiopathic chronic pancreatitis in Chinese patients. Dig Liver Dis. 2013;45:38–42. 23017645 10.1016/j.dld.2012.08.008

[24] Halangk W Wartmann T Sahin-Tóth M Nowack B Mayerle J Lerch MM. Preferential cleavage by cathepsin L of N34S-SPINK1 in comparison to the wild-type inhibitor. Pancreatology. 2016;13:S5.

[25] Boulling A Witt H Chandak GR. Assessing the pathological relevance of SPINK1 promoter variants. Eur J Hum Genet. 2011;19:1066–73. 21610753 10.1038/ejhg.2011.79 PMC3190254

[26] Boulling A Le Maréchal C Trouvé P Raguénès O Chen JM Férec C. Functional analysis of pancreatitis-associated missense mutations in the pancreatic secretory trypsin inhibitor (SPINK1) gene. Eur J Hum Genet. 2007;15:936–42. 17568390 10.1038/sj.ejhg.5201873

[27] Hassan Z Mohan V Ali L. SPINK1 is a susceptibility gene for fibrocalculous pancreatic diabetes in subjects from the Indian subcontinent. Am J Hum Genet. 2002;71:964–8. 12187509 10.1086/342731 PMC378551

[28] Kolly A Shivaprasad C Pulikkal AA Atluri S Sarathi V Dwarakanath CS. High prevalence of serine protease inhibitor Kazal type 1 gene variations detected by whole gene sequencing in patients with fibrocalculous pancreatic diabetes. Indian J Endocrinol Metab. 2017;21:510–4. 28670531 10.4103/ijem.IJEM_116_17 PMC5477435

[29] Sun C Liu MY Liu XG. Serine protease inhibitor Kazal type 1 (SPINK1) c.194 + 2T > C mutation may predict long-term outcome of endoscopic treatments in idiopathic chronic pancreatitis. Medicine (Baltim). 2015;94:e2046. 10.1097/MD.0000000000002046 PMC5058975 26632706

[30] Kume K Masamune A Mizutamari H. Mutations in the serine protease inhibitor Kazal type 1 (SPINK1) gene in Japanese patients with pancreatitis. Pancreatology. 2005;5:354–60. 15980664 10.1159/000086535

[31] Kume K Masamune A Kikuta K Shimosegawa T. [-215G>A; IVS3 + 2T>C] mutation in the SPINK1 gene causes exon 3 skipping and loss of the trypsin binding site. Gut. 2006;55:1214. 10.1136/gut.2006.095752 PMC1856255 16849362

[32] Melki G Laham L Karim G. Chronic pancreatitis leading to pancreatogenic diabetes presenting in diabetic ketoacidosis: a rare entity. Gastroenterology Res. 2019;12:208–10. 31523331 10.14740/gr1203 PMC6731042

[33] Bhat JA Bhat MH Misgar RA. The clinical spectrum of fibrocalculous pancreatic diabetes in Kashmir valley and comparative study of the clinical profile of fibrocalculous pancreatic diabetes and type 2 diabetes mellitus. Indian J Endocrinol Metab. 2019;23:580–4. 31803601 10.4103/ijem.IJEM_297_19 PMC6873263

[34] Unnikrishnan R Mohan V. Fibrocalculous pancreatic diabetes. Curr Diab Rep. 2020;20:19. 32277298 10.1007/s11892-020-01303-1

